# Retraction: Ultrasonic irradiation and SonoVue microbubbles-mediated RNA interfering targeting PRR11 inhibits breast cancer cells proliferation and metastasis but promotes apoptosis

**DOI:** 10.1042/BSR-2020-1854_RET

**Published:** 2024-09-10

**Keywords:** apoptosis, metastasis, microbubble, proliferation, Proline-rich protein 11, ultrasound

This article is being retracted from *Bioscience Reports* at the request of the authors. The authors were contacted by the Journal regarding concerns raised by a reader in relation to the appearance of Western blots in their paper. The authors responded that they were surprised that the paper had been published because the article processing fee was not paid and therefore they did not complete the publication process. The authors state that their paper was not ready for publication and there are outstanding issues that need to be addressed in the work. The authors wish to retract their article. The Editor-in-Chief and Editorial Board agree with the retraction. The Journal apologises for any inconvenience caused.

